# Prevalence and associated factors of eating disorders’ risk among Alexandria University medical students

**DOI:** 10.1186/s12889-026-27879-z

**Published:** 2026-06-03

**Authors:** Rana Abdelmohsen Hagag, Gihan Ismail Gewaifel, Soha Abd Ellatif Ibrahim, Rana Hassan Emara, Hend Mostafa Ali Ali

**Affiliations:** 1 Faculty of Medicine, Department of Community Medicine and Public Health, Alexandria University, Alexandria, Egypt; 2 Faculty of Medicine, Department of Neuropsychiatry, Alexandria University, Alexandria, Egypt; 3 Department of Nutrition, High Institute of Public Health, Alexandria University, Alexandria, Egypt

**Keywords:** Alexandria University, Associated factors, Eating disorders, Medical students, Prevalence

## Abstract

**Background:**

Eating disorders (EDs) are a group of mental health conditions involving aberrant eating behaviors and psychiatric symptoms, substantially affecting quality of life, physical and psychosocial functioning, particularly among medical students due to academic stress, transitional life phases, and lifestyle factors. Despite growing global awareness, EDs remain underdiagnosed in Arab regions.

**Methods:**

A cross-sectional study was conducted among 372 medical students during the academic year 2024–2025, using stratified random sampling. Data were collected via a self-administered questionnaire assessing sociodemographic, behavioral factors, medical, and family history of EDs, as well as the Eating Attitudes Test (EAT-26), and the Depression, Anxiety, and Stress Scale (DASS-21).

**Results:**

The prevalence of EDs’ risk was 21.8%, with most encountered disordered eating behaviors being excessive exercise (37.4%), followed by binge eating (14.8%), intentional vomiting (6.2%), and lastly laxatives or diuretics use (5.6%). Additionally, 70.7% of the students screened positively for depression, while 66.4% for anxiety, and 56.5% for stress. Independent predictors of EDs’ risk included female gender, clinical academic year, living alone, smoking, prior weight loss attempts, family history of underweight or malnutrition, and higher levels of depression and stress. Other factors, such as family income, obesity, and anxiety, were associated with EDs in univariate but not multivariate analyses. Conversely, Age, marital status, physical activity, and unsupervised psychiatric drug use showed no significant association.

**Conclusion:**

A substantial proportion of medical students exhibit risk of EDs and psychological distress. Targeted screening, mental health support, and integration of nutrition and stress management into the curriculum are essential to mitigate EDs’ risk in this vulnerable group.

## Introduction

Eating disorders (EDs) are a group of mental health conditions involving aberrant eating behaviours and psychiatric symptoms, often resulting in weight fluctuations and social impairment. They can substantially affect quality of life and daily functioning. [1]

These disorders, according to the latest version of the Diagnostic and Statistical Manual of Mental Disorders, fifth edition, text revision (DSM-5_TR), published in 2022 [2] and the latest version of the International Classification of Diseases, 11th Revision (ICD-11) [3] into several categories. Specified eating disorders include anorexia nervosa (AN; ICD-11 code: 6B80), characterized by restriction of energy intake and an intense fear of weight gain; bulimia nervosa (BN; ICD-11 code: 6B81), defined by recurrent episodes of uncontrolled binge eating followed by compensatory behaviors such as purging; and binge eating disorder (BED; ICD-11 code: 6B82), which involves recurrent binge eating in the absence of AN or BN. Additional categories include other specified feeding or eating disorders (OSFED; ICD-11 code: 6B8Y), encompassing conditions such as atypical anorexia nervosa, low-frequency or short-duration bulimia nervosa and binge eating disorder, and purging disorder, as well as unspecified feeding or eating disorder (UFED; ICD-11 code: 6B8Z), which applies to clinically significant disturbances that do not meet full diagnostic criteria for the previously mentioned categories.

Eating disorders are global phenomena, and their prevalence is rising worldwide. A systematic literature review found that the point prevalence of EDs ranged from 6.5% to 36% with an average mean of 19.4% for women. For men, the point prevalence ranged from 3.6% to 27.1% with an average mean of 13.8% [4]. 

EDs have a substantial physical and psychological impact, which can be assessed by measuring the QoL of EDs patient. It was found that EDs significantly affect Health Related Quality of Life (HRQoL), especially regarding psychological aspects of health. Evidence suggests that individuals suffering from EDs have impaired HRQOL not only in comparison to healthy individuals but also in comparison to other psychiatric patients, such as those suffering from major depression [5]. The low HRQoL of EDs patients is explained by their struggles in their relationships, social lives, and physical mobility, whether their BMI is controlled or not [6, 7]. Besides that, EDs not only worsen QOL of patients but also worsen that of their family, siblings, and caregivers [8, 9]. 

Furthermore, EDs are associated with different and significant physical and mental morbidities that can continue throughout patients’ lives or can lead to their death. Thus, prevention, early detection, and treatment are of extreme importance. Evidence showed that patients who didn’t receive the proper treatment have a significant risk for poor physical [10, 11]., mental outcome [12, 13]. and a significant risk of committing suicide in comparison to those who were successfully treated [11, 14]. 

Preventive measures and proper treatment of all ED cases are essential to save their lives. It is estimated that Prevention of ED cases can avoid an estimated mean of 213 deaths per 100,000 population. Proper treatment can avoid an estimated average of 70.5 deaths per 100,000 population. This emphasizes the importance of early detection, prevention, and control of EDs [15]. 

This is especially important among medical students who are considered a vulnerable group due to their age and the high level of stress, anxiety, and burnout that they experience, which makes them susceptible to mental health diseases, including eating disorders [16]. 

Thus, there is a need to conduct a study on the prevalence and associated factors of EDs among Alexandria University medical students.

## Methods

### Study design and setting

This cross-sectional study was conducted to screen eating disorders (EDs) among medical students at Alexandria University and to examine their association with sociodemographic and lifestyle factors, as well as depression, anxiety, and stress.

The study was carried out during the academic year 2024–2025 at the Faculty of Medicine, Alexandria University, Egypt.

### Study population and eligibility criteria

The study targeted Egyptian undergraduate medical students of both genders from the first to the fifth academic year. Eligible participants were students aged 18–25 years who were registered in the national undergraduate program, agreed to participate, and submitted a completed questionnaire. Students were excluded if they did not submit a completed questionnaire or reported psychiatric disorders other than eating disorders, depression, anxiety, or stress.

### Sampling technique and sample size

A stratified random sampling technique with proportional allocation was applied. The study population was first stratified by academic year (first to fifth year), and the total sample size was proportionally distributed across strata. Within each academic year, students are organized into teaching subgroups (“rounds”). Three rounds were randomly selected from each academic year, and within each selected round, students were randomly recruited until the required sample size for each stratum was achieved.

The sample size was calculated using the Epi Info-7 program [17] assuming a 95% confidence level, 80% power, and an expected prevalence of eating disorders among medical students of 33% [18]. The minimum required sample size was estimated at 340 participants. To account for an anticipated non-response rate of approximately 11–12% (40 participants), the sample size was increased to 380 participants.

### Data collection

A total of 380 anonymous self-administered questionnaires were distributed. Official approval to conduct the study was obtained from the Institutional Research Ethics Committee in the Faculty of Medicine, Alexandria University, before the study was conducted (IBR no: 00012098).

Participation of students was voluntary. After explaining the aim and importance of the study to the participants, informed consent was taken from all participants; they could withdraw any time. No bias was inflicted towards the students who refused to participate in the study, either in the quality of communication or the learning process.

### Study tools

Data were collected using a semi-structured questionnaire consisting of the following components:

#### Sociodemographic and lifestyle questionnaire

A pre-designed self-administered questionnaire was used to collect data on:

- Sociodemographic characteristics: age (years), academic year, gender, marital status, living arrangement, and family income.
- Lifestyle characteristics: participation in physical activity, including type, frequency, and duration. According to the Centers for Disease Control and Prevention (CDC) [19], physical activity was categorized as light, moderate, or vigorous. Smoking status was assessed and classified based on CDC definitions [20] into non-smoker, ex-smoker, and current smoker. Information on drug abuse (cannabis, heroin, cocaine, or others), alcohol consumption or abuse, and misuse of psychiatric drugs without a medical prescription was also collected.
- Weight management history: attempts to lose weight, methods used, and history of seeking nutritional therapy.
- Family history: eating disorders, obesity, and underweight-related conditions (e.g., cachexia, sarcopenia, etc.).
- Psychological history: history of eating disorders, depression, anxiety, stress, and other psychological disorders.

#### Eating Attitudes Test (EAT-26):^[21, 22^

The EAT-26 is a widely used standardized self-report tool for screening eating disorder symptoms. It consists of three subscales and five additional behavioral questions assessing eating disorder–related behaviors over the previous six months, including binge eating, self-induced vomiting, use of laxatives or other pills, excessive exercise (> 60 min), and weight loss of 9 kg or more. Self-reported weight and height were collected to calculate body mass index (BMI) [23]. 

The EAT-26 is a valid and reliable instrument that has been translated into multiple languages, including Arabic, which was used in the questionnaire. A previously validated Arabic version was used in this study; it was developed through forward–backward translation and tested among Egyptian university students, demonstrating acceptable reliability (Cronbach’s α = 0.728) and validity. These findings support its suitability for the present study population. [24]

A cut-off score of ≥ 20 was used to indicate a significant risk of eating disorders. This cut-off has been validated in both clinical and non-clinical populations [25]. 

#### Depression, Anxiety, and Stress Scale-21 (DASS-21):^[26, 27^

The Arabic version of DASS-21 is a self-report instrument designed to assess the emotional states of depression, anxiety, and stress. It demonstrates excellent internal consistency, with all items contributing comparably to overall reliability.

### Pilot study

A pilot study was conducted on 33 randomly selected Egyptian medical students from the national program. Data from the pilot study were used to refine the semi-structured questionnaire, but were not included in the final data analysis.

### Data processing and analysis

The collected data were revised, coded, and fed to the computer using the Statistical Package for Social Science (SPSS/PCT) program (version 23) [17]. Appropriate descriptive and inferential statistical analyses were conducted. The statistical test used were chi-square to test the association between categorical variables, and the Mann-Whitney test for non- normally distributed quantitative variables.

#### Regression analysis

Variables that were significant in the bivariate analysis were included in the regression model after excluding multicollinearity, as assessed using the correlation matrix. The model was then constructed using the forward selection method. The Hosmer–Lemeshow test significance was reported to assess the goodness of fit of the model. Nagelkerke R² was reported to demonstrate the contribution of significant variables in explaining variation in the outcome. Reference categories were provided as footnotes below the regression Table (8).

## Results

A total number of 380 questionnaires were submitted, out of which 372 questionnaires were valid and 8 questionnaires were excluded.

Nearly half of the studied students were female (53.5%), with a mean age of 20.7 ± 1.7 years, ranging from 18 to 25 years. The distribution across academic years was nearly equal. Most students were single (94%).

Regarding living arrangements, 64.5% resided with their families, 25.3% lived on campus, 6.7% lived alone, and 3.5% lived with relatives. Approximately half of the participants (50.5%) reported having more than sufficient family income, as shown in Table 1.

**Table 1:** Distribution of the studied medical students (n=372) at Alexandria University according to their Sociodemographic characteristics, 2024

Sociodemographic characteristics		Total (n=372)		
		no.		%
Gender				
Male		173	46.5	
Female		199	53.5	
Age (years)				
18		47	12.6	
19		59	15.9	
20		78	21.0	
21		55	14.8	
22		64	17.2	
23		63	16.9	
24		5	1.3	
25		1	0.3	
Min – Max		18 – 25		
Mean ± SD		20.7 ± 1.7		
Median (IQR)		21 (3)		
Academic year				
Preclinical	First	78	21	
	Second	72	19.4	
	Third	71	19.1	
clinical	Fourth	71	19.1	
	fifth	80	21.5	
Marital Status				
Single		351	94.4	
Engaged		18	4.8	
Married		3	0.8	
Living arrangement				
Alone		25	6.7	
With family		240	64.5	
With relatives		13	3.5	
campus		94	25.3	
Family income				
Not enough		15	4	
Barely enough		169	45.5	
Enough and saving		188	50.5	

Table 2 illustrates that only 31.2% of the surveyed students were physically active, with the majority engaging in moderate activity (59.5%). The mean weekly physical activity duration was 234 minutes (range: 30–720 minutes). Most students were non-smokers (96.2%). Nearly all students (98%) reported no current or previous drug or alcohol abuse; however, 1.3% had past abuse and 0.3% were currently abusing either cannabis (50%), alcohol (16.7%), or both (33%). A small proportion (2.5%) reported psychiatric drug use without consultation, including 2.2% with past misuse and 0.3% with current misuse. Regarding psychiatric history, 46.2% reported no psychiatric disorders, 38.4% were currently affected, and 15.3% had previous disorders. Positive family history of eating disorders was reported by 12.6%, underweight-related conditions by 7%, and obesity by 25.5% of the participants.

**Table 2:** Distribution of the studied students (n=372) at Alexandria Faculty of Medicine according to personal lifestyle characteristics, history of psychological disorders, and family history, 2024

Lifestyle characteristics	Total (n=372)		
	no.		%
Exercise(n=372)			
No	256	68.8	
Yes	116	31.2	
Level of physical activity (n=116)			
Light	8	6.9	
Moderate	69	59.5	
vigorous	39	33.6	
Duration of sport practice minutes per week (n=116)			
30-60	10	8.6	
60 - 120	30	25.9	
120 -180	26	22.4	
180 – 240	7	6	
More than 240	43	37.1	
Min – Max	30 – 720		
Mean ± SD	234.1 ± 161.7		
Median (IQR)	180 (225)		
Smoking history(n=372)			
Non-smoker	358	96.2	
Ex-smoker	2	0.5	
Currently smoker	12	3.2	
Drug and alcohol abuse (n=372)			
No	366	98.4	
previously abused	5	1.3	
currently abuse	1	0.3	
Type of Drug /alcohol abuse (n=6)			
alcohol only	1	16.7	
Cannabis only	3	50.0	
Alcohol & Cannabis	2	33.3	
Psychiatric drug uptake without consultation (n=372)			
No	363	97.6	
previously abused	8	2.2	
currently abuse	1	0.3	
Category of medicine abused (n=9)			
antidepressant	2	22.2	
antiepileptic	1	11.1	
antipsychotic	1	11.1	
sedative	3	33.3	
stimulants	2	22.2	
History of psychiatric disorders (n=372)			
No	172	46.2	
Yes, in the past	57	15.3	
Yes, currently	143	38.4	
Family history of eating disorders (n=372)			
No	325	87.4	
Yes	47	12.6	
Family history of underweight diseases (n=372)			
No	346	93	
Yes	26	7.0	
Family history of obesity (n=372)			
No	277	74.5	
Yes	95	25.5%	

Approximately 28.8% of students reported seeking medical consultation for weight- or eating-related concerns, primarily for weight loss (54.2%). More than half of the participants (58.4%) had previously attempted weight reduction, of whom 23.7% tried once, and 34.7% reported multiple attempts. Combined dietary modification and exercise was the most commonly adopted method (40.6%). The mean BMI of the studied sample was 24.6 kg/m² (range: 15.6–41.8 kg/m²). While 57.0% of students were within the normal BMI category, 4.3% were underweight, and 38.7% were either overweight (26.3%) or obese (12.4%), as shown in Table 3.

**Table 3:** Distribution of the studied medical students (n=372) at Alexandria University according to weight management and dieting history and their Body Mass Index (BMI), 2024

Weight management history	Total (n=372)		
	no.		%
History of seeking nutritional therapy for weight/eating problems (n=372)			
No	265	71.2	
Yes	107	28.8	
Purpose of seeking medical advice (n=107)			
Losing weight	58	54.2	
Gaining weight	36	33.6	
others	13	12.2	
Weight Loss trial (n=372)			
No	155	41.6	
Yes, only once	88	23.7	
Yes, more than once	129	34.7	
Method used to lose weight (n=217)			
Diet	69	31.8	
exercise	52	23.9	
medicine	1	0.5	
diet and exercise	88	40.6	
diet, exercise &medicine	5	2.3	
diet, exercise, medicine & bariatric surgery	2	0.9	
BMI(Kg/m^2^)			
Normal (BMI18.5-24.9)	212	57.0	
Underweight (BMI<18.5) Over-weight (BMI 25-29.9) obese (BMI ³ 30)	169946	4.326.312.4	
MIN – Max	15.6 – 41.8		
Mean ± SD	24.6 ± 4.5		
Median (IQR)	23.8 (5.7)		

Table 4 demonstrates that the overall EAT_26 score ranged from 0 to 58, with a mean of 12.9.

**Table 4:** Distribution of the studied medical students (n=372) at Alexandria University according to their total score and score in each subscale of Eating Attitude Test-26 (EAT-26) questionnaire, 2024

Subscales of EAT_26	Total (n=372)
	no.
Dieting subscale	
Min-Max	0 - 35
Mean ± SD	6.9 ± 6.8
Median (IQR)	5 (8)
Bulimia and food preoccupation scale	
Min-Max	0 - 12
Mean ± SD	1.5 ± 2.3
Median (IQR)	0 (2)
Oral control scale	
Min-Max	0 - 21
Mean ± SD	4.6 ± 4.1
Median (IQR)	3 (5)
Overall EAT-26 score	
Min-Max	0 - 58
Mean ± SD	12.9 ± 9.2
Median (IQR)	11 (11)

**Fig. 1:**
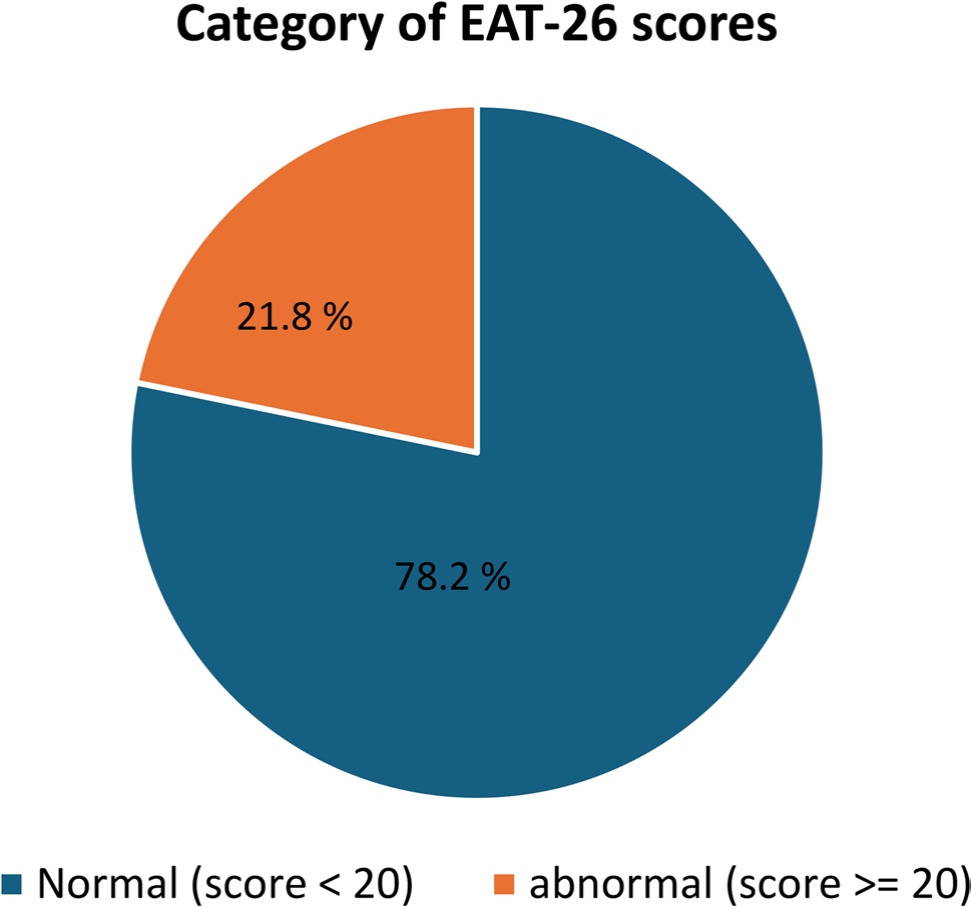
Prevalence of eating disorders’ risk among the studied medical students (n=372) at Alexandria University, 2024

Figure 1 demonstrates that 21.8% of participants scored 20 or higher in the EAT-26 scale, indicating the presence of potential EDs. However, the majority of participants (78.2%) had normal scores.

The most frequently experienced abnormal behaviour in the past 6 months was excessive exercise (37.4%), followed by bingeing (14.8%). In contrast, classic compensatory purging behaviours like self-induced vomiting (6.2%) and misuse of laxatives/diet pills/diuretics (5.6%) were the least common. Additionally, 18.3% of the participants in the study reported significant weight loss of more than 9 kg in the past 6 months.

Table 5 demonstrates that the total scores on each DASS-21 subscale among the studied medical students showed that the stress subscale had the highest mean score (18.3 ± 10.4), followed by depression (16.2 ± 10.6), and then anxiety (13.0 ± 9.8).

**Table 5:** Distribution of the studied medical students (n=372) at Alexandria University according to grades of depression, anxiety, and stress, 2024

Category of DASS-21	Total (n=372)	
	no.	%
Depression subscale		
Normal	109	29.3
Abnormal:	263	70.7
Mild	56	15.1
Moderate	93	25.0
Severe	44	11.8
Extremely severe	70	18.8
Anxiety subscale		
Normal	125	33.6
Abnormal:	247	66.4
Mild	33	8.9
Moderate	76	20.4
Sever	37	9.9
Extremely sever	101	27.2
Stress subscale		
Normal	162	43.5
Abnormal:	210	56.5
Mild	44	11.8
Moderate	73	19.6
Sever	55	14.8
Extremely sever	38	10.2

On studying the distribution of medical students across severity categories for each subscale of the DASS-21 (Depression, Anxiety, and Stress), it was found that 70.7% of the students experienced depressive symptoms ranging from mild to extremely severe.

Regarding the anxiety scale, a total of 66.4% of students scored in the abnormal range, with 27.2% classified as experiencing extremely severe anxiety, which is the highest single category across all subscales.

Regarding the stress scale, more than half (56.5%) experienced abnormal stress levels.

Table 6 demonstrates that eating disorders’ risk was significantly higher among females, among clinical students (27.2%) compared to preclinical students (18.1%), and also among those living alone (48%). Family income also showed a statistically significant association, where Students with insufficient or barely sufficient income showed the highest prevalence (26.6%).

**Table 6:** Distribution of the studied medical students (n=372) at Alexandria University according to their Sociodemographic characteristics and eating disorders’ risk, 2024

Sociodemographic Characteristic	EAT-26				Test of significance (p value)
	Normal (<20)		Abnormal (³20)		
	(n=291)	(%)	(n=81)	(%)	
Gender					
Male	146	84.4	27	15.6	χ2(df)=7.222(1),P=0.007**
Female	145	72.9	54	27.1	
Age					
Mean ± SD	20.6 ± 1.7		20.9 ± 1.8		U= 10665P=0.18
Median (IQR)	20 (3)		21 (3.5)		
Academic year					
Preclinical	181	81.9	40	18.1	χ2(df)= 4.316(1),P=0.03*
Clinical	110	72.8	41	27.2	
Marital Status					
Single	273	77.8	78	22.2	χ2(df)= 1.15(2),P= 0.560
Engaged	15	83.3	3	16.7	
Married	3	100.0	0	0	
Living Arrangement					
Alone	13	52.0	12	48.0	χ2(df)= 11.382(3),P=0.01**
With Family	194	80.8	46	19.2	
With relatives	11	84.6	2	15.4	
Campus	73	77.7	21	22.3	
Family income					
Not enough / Barely enough	135	73.4	49	26.6	χ2(df)=5.041(1),P= 0.02*
Enough and saving	156	83.0	32	17.0	

Preclinical = first, second, and third grades, Clinical = fourth and fifth grades. *Significant at p value ≤0.05, **significant at p value ≤0.01, χ^2^(df): Chi square test(degree of freedom), U= Mann–Whitney U

Table 7 showed that EDs’ risk was significantly higher among current smokers, and also among students with history of drug or alcohol. Additionally, students with a history of psychiatric disorders had a significantly higher prevalence, where it represented 29.4% in current cases and 28.1% in past cases. Similarly, students who had a positive family history of EDs, underweight diseases, and obesity also had significantly higher prevalence, which represented 36.2%, 42.3%, and 34.7% respectively. Additionally, seeking nutritional therapy for weight or eating problems (33.6%) and attempting weight loss more than once (60.5%) were associated with EDs’ risk. Among weight loss methods, those using combined strategies including diet, exercise, and bariatric surgery showed the highest prevalence (100%). Furthermore, students with abnormal BMI values were more likely to have EDs (30%) than those with normal BMI (15.6%).

**Table 7:** Distribution of the studied medical students (n=372) at Alexandria University according to their lifestyle characteristics, medical history, Body Mass Index (BMI), and eating disorders’ risk, 2024

Independent Variables	EAT-26	Normal (score<20)		Abnormal (score³20)		Test of significance (p value)
		(n=291)	(%)	(n=81)	(%)	
Lifestyle characteristics						
Exercise(n=372)						
No		195	76.2	61	23.8	χ2(df)= 2.03(1),P= 0.15
Yes		96	82.8	20	17.2	
Smoking history(n=372)						
Non-smoker		284	79.3	74	20.7	χ2(df)= 10.23(2),P=0.006**
Ex-smoker		2	100.0	0	0	
Current smoker		5	41.7	7	58.3	
Drug and alcohol abuse (n=372)						
no		290	79.2	76	20.8	χ2(df)=13.76(2),P=0.001**
previously abused		1	20	4	80.0	
currently abuse		0	0	1	100.0	
Psychiatric drug uptake without consultation (n=372)						
no		286	78.8	77	21.2	χ2(df)= 4.09(2),P=0.13
previously abused		4	50.0	4	50.0	
currently abuse		1	100.0	0	0	
Psychological Disorders history						
History of psychiatric disorders (n=372)						
No		149	86.686.6	23	13.4	χ2(df)= 13.3(2),P= 0.001**
Yes, in the past		41	71.9	16	28.1	
Yes, currently		101	70.6	42	29.4	
Family history						
Family history of eating disorders		30	63.8	17	36.2	χ2(df)= 6.546(1),P=0.011*
Family history of underweight diseases		15	57.7	11	42.3	χ2(df)= 6.919(1),P=0.009**
Family history of obesity		62	65.3	33	34.7	χ2(df)= 12.586(1),P < 0.001*
Weight management history						
History of seeking nutritional therapy for weight/eating problem (n=372)						
No		220	83.0	45	17.0	χ2(df)= 12.43(1),P < 0.001**
Yes		71	66.4	36	33.6	
Purpose of seeking medical advice (n=107)						
Losing weight		34	58.6	24	41.4	χ2(df)= 3.71(2),P=0.16
Gaining weight		28	7.8	8	22.2	
others		9	69.2	4	30.8	
Weight Loss trial (n=372)						
No		137	47.1	18	22.2	χ2(df)= 31.07(2),P < 0.001**
Yes, once		74	25.4	14	17.3	
Yes, more than once		80	27.5	49	60.5	
Method used to lose weight (n=217)						
diet		47	68.1	22	31.9	χ2(df)= 15.23(5),P=0.009**
exercise		46	88.5	6	11.5	
medicine		1	100.0	0	0	
diet exercise		57	64.8	31	35.2	
diet, exercise &medicine		3	60.0	2	40.0	
diet, exercise, medicine & bariatric surgery		0	0	2	100.0	
BMI(Kg/m^2^)						
Normal BMI (BMI18.5-24.9)		179	84.4	33	15.6	χ2(df)= 11.15(1),P= 0.001**
Abnormal BMI		112	70.0	48	30.0	

**Fig. 2:**
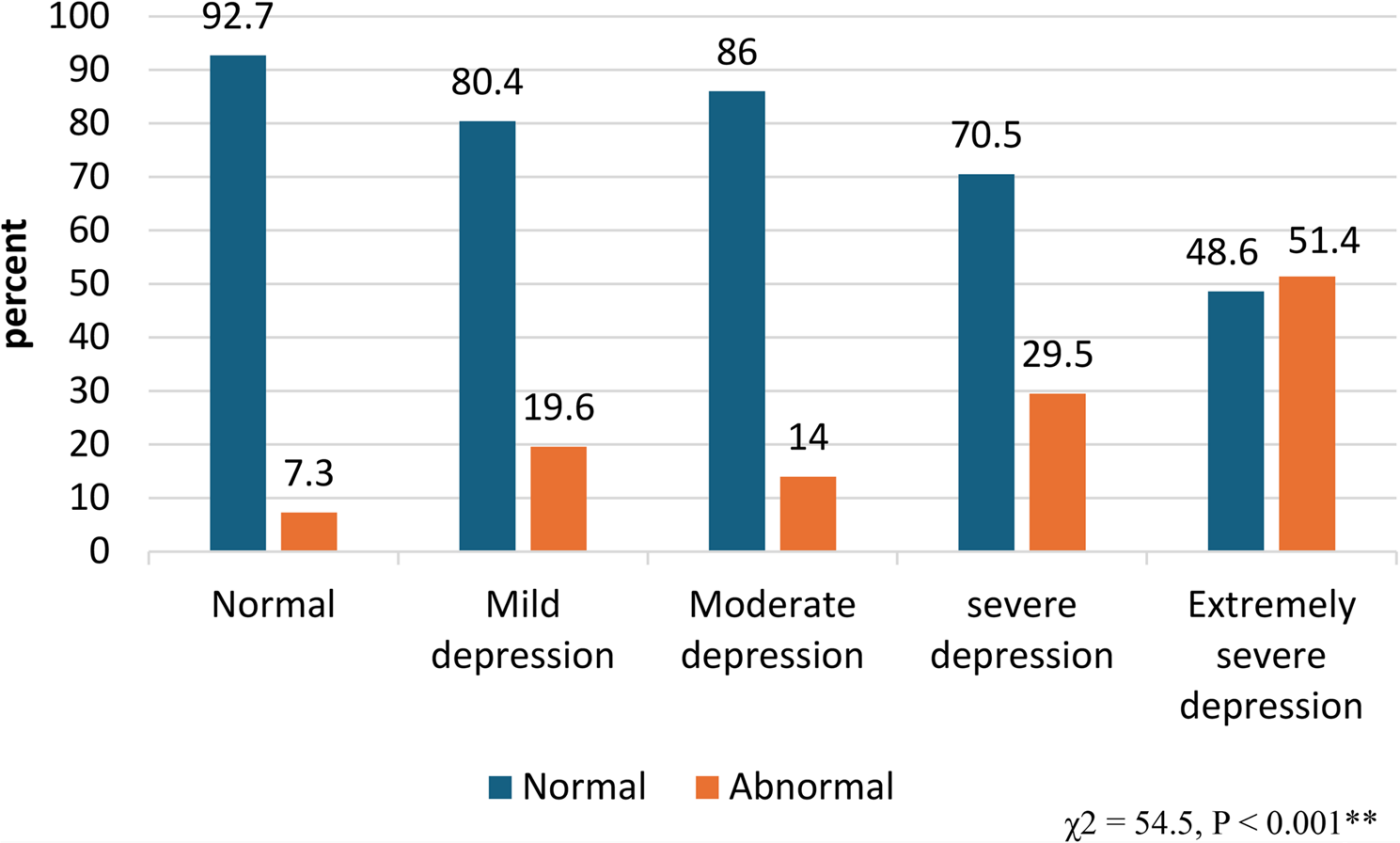
Distribution of depression severity grades (assessed by the DASS-21) among studied medical students (n=372) at Alexandria University and eating disorders’ risk (measured by EAT-26), 2024

Figure 2 showed that Students classified as having extremely severe depression exhibited the highest prevalence of EDs’ risk. The association was highly statistically significant (χ² = 54.5, p < 0.001).

**Fig. 3:**
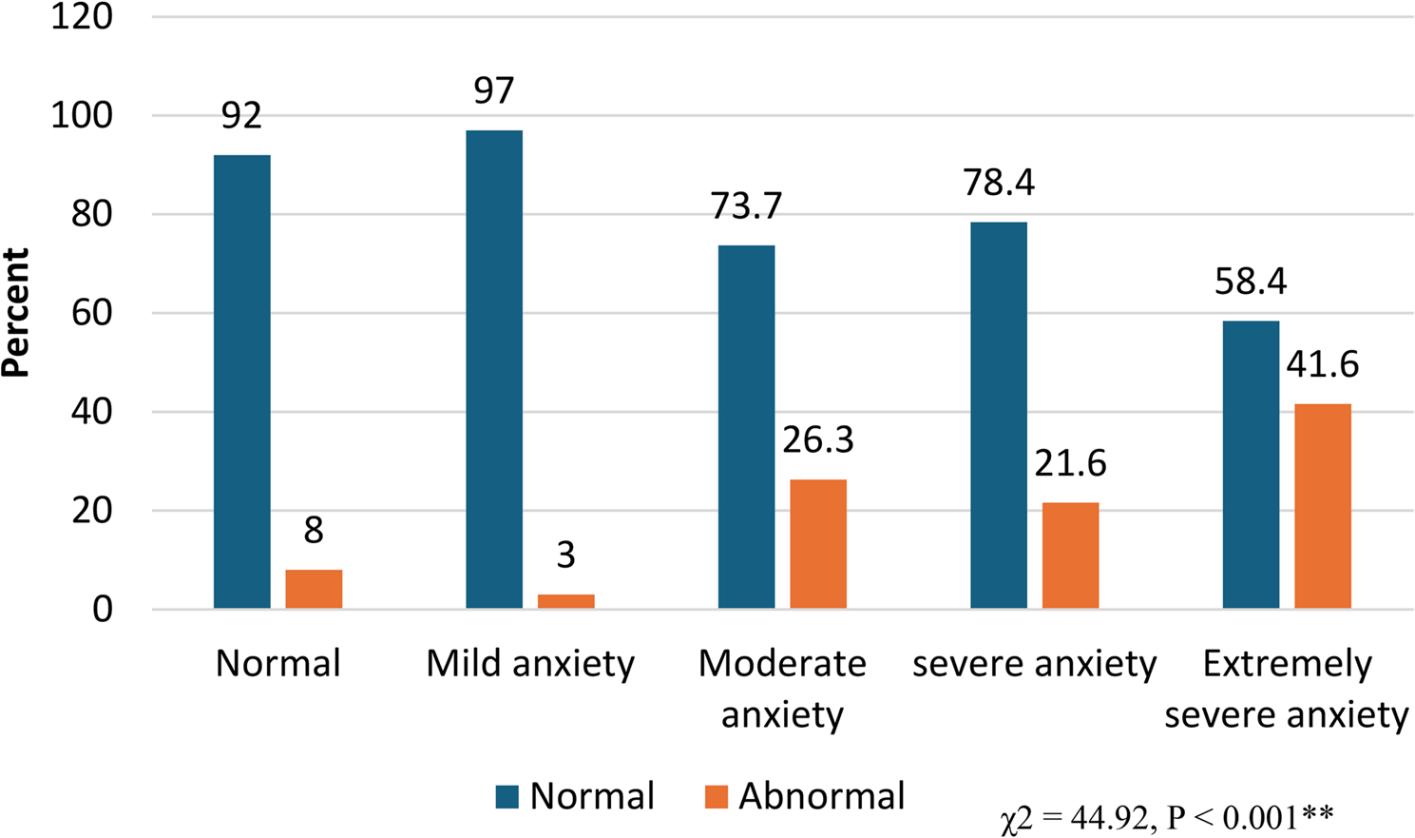
Distribution of Anxiety severity grades (assessed by the DASS-21) among studied medical students (n=372) at Alexandria University and eating disorders’ risk (measured by EAT-26), 2024

Students classified as having extremely severe anxiety were more likely to have EDs (χ² = 44.9, *p* < 0.001), as shown in Fig. 3.

**Fig. 4:**
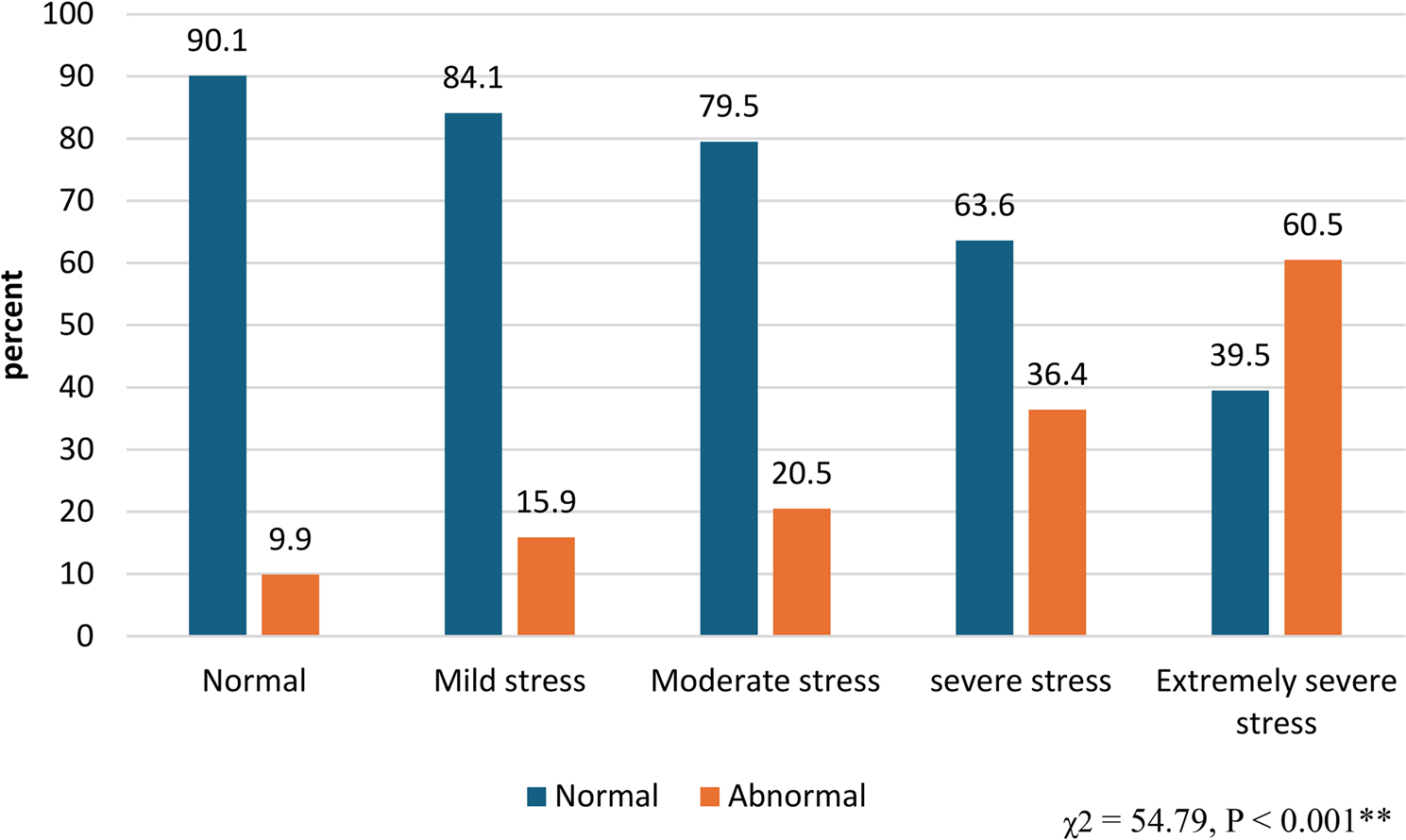
Distribution of stress severity grades (assessed by the DASS-21) among studied medical students (n=372) at Alexandria University and eating disorders’ risk (measured by EAT-26), 2024

Students classified as having extremely severe stress exhibited the highest prevalence of EDs. The association was highly statistically significant (χ² = 54.8, *P* < 0.001), as shown in Fig. 4.

Logistic regression analysis identified several independent predictors of eating disorders among medical students (Table 8). In the multivariate model (R² = 0.393, *p* < 0.001), female gender (*p* = 0.007), clinical academic grade (*p* = 0.020), living alone (*p* = 0.013), current smoking (*p* = 0.033), positive family history of underweight (*p* = 0.008), and previous weight loss attempts, whether once (*p* < 0.001) or multiple times (*p* < 0.001), both depression and stress in all grades were significant predictors of EDs.

**Table 8:** Univariate and multivariate regression analysis of different independent factors and eating disorders

Independent predictors	Univariate regression				Multivariate regression P<0.001** R^2^= 0.393			
	p value	Exp. β	UL of CI	LL of CI	p value	Exp. β	UL of CI	LL of CI
Gender	0.008**	2.014	1.202	3.374				
Male gender					0.007**	0.417	0.221	0.786
Academic year								
Preclinical academic grades	0.039*	0.593	0.361	0.974	0.02*	0.483	0.261	0.893
Living Arrangement								
Living with family , relatives or on campus	0.002**	3.719	1.625	8.51	0.013*	3.724	1.321	10.496
Cigarette smoking								
non smoker	0.005**	0.185	0.057	0.599	0.033*	0.182	0.038	0.875
Negativefamily history of thinness and related diseases	0.01*	0.346	0.152	0.786	0.008*	0.213	0.068	0.664
Losing weight trial	<0.001**				<0.001**			
No trial to lose weight	<0.001**	0.215	0.117	0.393	<0.001**	0.181	0.087	0.375
Only one trial to lose weight	0.001**	0.309	0.158	0.605	<0.001**	0.234	0.106	0.519
Depression	<0.001**				0.018*			
No depression	<0.001**	0.105	0.047	0.236	0.017*	0.269	0.092	0.788
Mild/moderate depression	<0.001**	0.255	0.144	0.452	0.014*	0.394	0.188	0.827
Stress	<0.001**				0.026*			
No stress	<0.001**	0.127	0.066	0.246	0.012*	0.302	0.118	0.769
Sever/ extremely severe stress	<0.001**	0.269	0.145	0.499	0.03*	0.428	0.199	0.921
Family income								
Family income more than enough and saving	0.026*	1.769	1.072	2.922	0.108			
Drug Abuse								
No drug abuse	0.008**	0.052	0.006	0.455	0.188			
Positive family history of eating disorders	0.012*	0.433	0.225	0.833	0.523			
Family history of obesity and related diseases	<0.001**	2.539	1.503	4.291	0.095			
BMI								
Normal BMI	0.001**	0.430	0.26	0.711	0.286			
Anxiety	<0.001**				0.299			
No anxiety	<0.001**	0.153	0.073	0.319	0.138			
Mild / moderate anxiety	0.004**	0.420	0.233	0.757	0.682			

Reference categories used in regression for gender: male, academic year: clinical grades, living arrangement: living alone, cigarette smoking: current smoker, history of thinness: positive history, losing weight trial: more than one trial, depression: severe and extremely severe, stress: severe and extremely severe, Anxiety: severe and extremely severe, Family income: more than enough and saving, drug abuse: previously or currently abusing , family history of eating disorders: positive history, family history of obesity: positive history, BMI: abnormal BMI

In contrast, the variables such as family income, drug abuse, family history of eating disorders, family history of obesity-related diseases, abnormal BMI, and anxiety showed statistical significance in the univariate analysis but lost significance in the multivariate model (*p* > 0.05).

## Discussion

The prevalence of EDs’ risk among the studied sample, based on the cut-off score of the EAT-26 questionnaire, was found to be 21.8%. In comparison to other recent studies that have used a similar methodology conducted internationally, the prevalence of the present study was relatively higher. As the risk internationally was 10% in Spain [28], 11% in Malaysia [29], 13.9% in Brazil [30], and 14.9% in Uganda [31]. These differences may stem from variations in cultural perceptions of body image, dietary habits, and societal norms. In addition, emerging psychosocial influences such as social media exposure, online nutrition information, and weight-related self-stigma may further shape body dissatisfaction and maladaptive eating attitudes among young adults, potentially contributing to cross-cultural variation in eating disorder risk. [32]

Regionally, the prevalence of the present study more or less aligns with findings from Middle Eastern countries like Saudi Arabia (25.7%) [33], Palestine (28%) [34], and Iraq (28.7%) [35]. These consistent regional figures suggest a shared vulnerability possibly rooted in sociocultural values. However, the United Arab Emirates and Lebanon had a lower risk, with a prevalence of 14% [35]. and 16.5%. respectively [37]. 

Analysis of EAT-26 responses found that the most frequently experienced abnormal behaviour was excessive exercise for more than 60 min to lose weight, where 37.4% of the enrolled students scored positively, followed by binge eating behaviour, where positive responses represented 14.8%. Lastly, purging symptoms, where 6.5% of the study participants responded positively to practicing intentional vomiting, and 5.6% used diet pills, laxatives, and diuretics to control their weight and shape. A similar pattern was observed in other studies, but with higher frequencies [38]. 

The present study demonstrated a markedly high prevalence of psychological distress among medical students, with 70.7%, 66.4%, and 56.5% screening positive for depression, anxiety, and stress, respectively. These estimates are substantially higher than those reported in robust international evidence. For instance, a large systematic review and meta-analysis including 167 studies (*n* = 116,628) reported a pooled prevalence of depression or depressive symptoms of 27.2% among medical students, while a more recent individual participant data meta-analysis estimated even lower pooled prevalence at 18.1% when standardized cut-offs were applied [39].

At the regional level, studies using comparable tools have also reported lower estimates; for example, a study conducted among Egyptian medical students using the DASS-21 reported a prevalence of depression of 45.2%, which remains considerably lower than the 70.7% observed in the current study. The discrepancy may be explained by several methodological and contextual factors. First, the use of the DASS-21, a sensitive screening instrument, captures a broader spectrum of symptom severity, including mild and subclinical cases, thereby yielding higher prevalence estimates compared to diagnostic interviews. Second, variations in cut-off thresholds and classification criteria across studies significantly influence reported prevalence, as highlighted by the wide range of depression estimates (9.3%–55.9%) across different measurement approaches. Third, contextual factors specific to the study setting—such as the high academic burden of medical training, sociocultural pressures, and potential economic stressors—may further exacerbate psychological distress. Additionally, the reliance on self-reported data introduces the possibility of reporting bias, while the timing of data collection (e.g., proximity to examinations) may have contributed to transient elevations in stress and anxiety levels. Despite these considerations, the strong and highly significant associations observed between depression, anxiety, stress, and eating disorder risk are consistent with the broader literature, which identifies psychological distress as a central correlate of disordered eating behaviors through mechanisms such as emotional dysregulation and maladaptive coping. [40]

Additionally, the reliance on self-reported data introduces the possibility of response bias, including overreporting due to increased mental health awareness or situational stressors (e.g., academic workload at the time of data collection). Despite these considerations, Depression, Anxiety, and stress were found to have strong and highly statistically significant associations with EDs’ risk in both univariate and multivariate analyses (*p* < 0.001 in all). Furthermore, a strong link between the increasing severity of these mental disorders and the risk of EDs was observed. These findings align with different studies conducted regionally and internationally. Where the association was noted in a study conducted on Egyptian university students [40] and in another conducted on Egyptian youth [41]. A similar finding was also noted in a study conducted in Lebanon [37]. 

Furthermore, the result of the current study highlights the association between the risk of developing EDs and various sociodemographic characteristics among the studied medical students. These included gender, academic year, living arrangement, and family income. Notably, gender emerged as a significant associated factor, with a higher prevalence among females. This association was not only significant in univariate analysis (*p* = 0.008) but also in multivariate analysis (*p* = 0.007). This is consistent with results from different countries and settings [33, 34, 42]. 

Students in clinical grades showed more EDs’risk. This association was also significant in both univariate and multivariate analyses. Due to the stress levels over the years, as a result of course requirements, responsibilities, and self-demands, that make them vulnerable to mental disorders, including EDs [43]. 

Students who lived alone were most affected. This finding was prominent in a study that investigated the influence of loneliness on EDs, and found that loneliness not only showed higher psychopathology but also higher weight concerns and stress levels among EDs patients [44]. 

Moreover, family income was associated with EDs’ risk, with those reporting insufficient income at greater risk. This finding was also found in a study conducted on university students in a developing country like Bangladesh [45]. 

Regarding students’ habits, being a Smoker was significantly associated with EDs, this is consistent with a study on university students in Palestine, where EDs’ risk among them was significantly associated with smoking and depression [34]. 

Interestingly, positive family history has shown a significant association, where students who had a positive family history of EDs, thinness, and obesity were at higher risk. This is in line with previous evidence discussing genetic vulnerability and acquired behaviours as major risk factors for EDs development. Family members share unhealthy dietary habits, cultural values, and norms [46]. 

Additionally, an abnormal body mass index (BMI), whether underweight or overweight/obese, increases the risk of having EDs among the studied students, a finding that aligns with previous research, especially among individuals with obesity [33, 34, 40, 47, 48]. In parallel, students who had previously sought medical help for weight or eating problems, or had attempted weight loss trials, especially those with multiple attempts and had employed specific weight loss strategies such as dieting, were also significantly more likely to exhibit risk of EDs. These findings collectively highlight the interplay between BMI status, weight-control behaviours, and the risk of developing disordered eating patterns.

### Study limitations

Despite these important findings, several limitations should be considered when interpreting the results:

- The design of the current study was a cross-sectional design; thus, causality and temporal relationship cannot be established., the association could be assumed Therefore, future research with a longitudinal design is recommended.
- The study population was restricted to medical students, a relatively homogeneous and highly educated subgroup of young adults. This may limit the representativeness of the findings, and broader population-based studies are needed to better characterize the epidemiology of eating disorders (EDs).
- As the study was conducted in a single institution, the findings have limited external validity. They are most appropriately generalizable to medical students in similar academic and sociocultural contexts, and may not be applicable to students from other disciplines or to the general population.
- All the data in the current study were self-reported, so recall bias and response bias and inevitably existed.
- Social desirability bias cannot be excluded, as data were collected via self-reported measures. The anonymous nature of the questionnaire likely mitigated this effect by promoting honest disclosure of sensitive behaviors.

### Study strength

The sample size of 372 participants is considered adequate for cross-sectional research and provides sufficient statistical power to detect meaningful associations. In addition, the use of validated and widely accepted screening instruments (EAT-26 and DASS-21) strengthened the reliability and comparability of the findings.

## Conclusion 

The current study revealed a concerning prevalence of EDs among Alexandria medical students, with over one-fifth of participants identified as at risk based on the EAT-26 scale. ED risk was significantly associated with psychological distress, particularly depression, anxiety, and stress. This highlights the importance of integrating mental health and nutritional support within university settings. Other factors, including sociodemographic factors, lifestyle factors, and abnormal BMI, were significantly associated with ED risk. These findings point to the complex interplay of environmental, psychological, and biological influences on eating behaviors.

These findings highlight the need for an integrated approach within university settings that combines mental health support with nutritional guidance. In this context, the implementation of early screening strategies, comprehensive and multidisciplinary interventions, and culturally sensitive prevention programs is essential to mitigate ED risk and its potential impact on students’ well-being, academic performance, and future professional roles.

Furthermore, Longitudinal studies are recommended to explore the trajectory of eating disorders and mental health status over the duration of medical education. Moreover, future research should assess the effectiveness of implemented interventions and explore other potential risk factors, including media exposure, sleep patterns, and social media use.

## Data Availability

The datasets generated and/or analysed during the current study are not publicly available due to participant confidentiality and ethical restrictions, but are available from the corresponding author on reasonable request.

## Abbreviations

AN: Anorexia Nervosa
BED: Binge Eating Disorder
BMI: Body Mass Index
BN: Bulimia Nervosa
DASS-21: Depression, Anxiety, and Stress Scale – 21 items
CDC: Center of Disease And Control
DSM-5-TR: Diagnostic and Statistical Manual of Mental Disorders, Fifth Edition, Text Revision
EAT-26: The Eating Attitudes Test − 26 items
EDs: Eating Disorders
HRQOL: Health Related Quality of Life
ICD-11: International Classification of Diseases 11th Revision
OSFED: Other Specified Feeding or Eating Disorders
UFED: Unspecified Feeding or Eating Disorders
